# A pre-formed Pyrogenic Factor Released by Lipopolysaccharide Stimulated Macrophages

**DOI:** 10.1155/S0962935194000517

**Published:** 1994

**Authors:** A. R. Zampronio, M. C. C. Melo, C. A. A. Silva, I. R. Pelá, S. J. Hopkins, G. E. P. Souza

**Affiliations:** 1 Laboratory of Pharmacology Faculty of Pharmaceutical Sciences University of São Paulo Ribeirão Preto S.P. 14049-903 Brazil; 2 University of Manchester Rheumatic Diseases Centre Hope Hospital Salford M6 8HD UK

## Abstract

The aim of this study was to investigate the pyrogenic activity of factor(s) released by rat peritoneal macrophages following a brief stimulation with LPS. The effect of this factor on the number of circulating leukocytes and serum Fe, Cu and Zn levels, was also evaluated. The possibility that the content of interleukin (IL)-1β, IL-6 and tumour necrosis factor (TNF) in the supernatant could explain the observations was investigated. Supernatant produced over a period of 1 h by peritoneal macrophages, following a 30 min incubation with LPS at 37°C, was ultrafiltered through a 10 000 MW cut-off Amicon membrane, sterilized, and concentrated 2.5, 5, 10 and 20 times. The intravenous (i.v.) injection of this supernatant induced a concentration-dependent fever in rats with a maximal response at 2 h. The pyrogenic activity was produced by macrophages elicited with thioglycollate and by resident cells. The supernatants also induced neutrophilia and reduction in Fe and Zn 6 h after the injection. Absence of activity in boiled supernatants, or supernatants from macrophages incubated at 4°C with LPS, indicates that LPS was not responsible for the activity. *In vitro* treatment with indomethacin (Indo), dexamethasone (Dex), or cycloheximide (Chx) did not modify the release of pyrogenic activity into the supernatant or its effects on the reduction in serum metal levels. Although Chx abolished the production of mediator(s) inducing neutrophilia, and Dex reduced the induction of IL-1β, TNF and IL-6, injection of the highest concentration of these cytokines detected in the supernatants did not induce fever. *In vivo* treatment with Dex, but not Indo, abolished the fever induced by the supernatant. These results suggest that macrophages contain pre-formed pyrogenic mediator(s), not related to IL-1β, IL-6 or TNF, that acts indirectly and independently of prostaglandtn. It also seems likely that the pyrogenic activity is related to the factor responsible for the reduction of serum Fe and Zn levels, but not the neutrophilia.


**Research Paper**

Mediators of Inflammation 3, 365–373 (1994)

### A pre-formed pyrogenic factor released by lipopolysaccharide stimulated macrophages

A. R. Zampronio,[1] M. C. C. Melo,[1] C. A. A. Silva,[1] I. R. Pelá,[1] S. J. Hopkins[2] and G. E. P. Souza[1,CA]

[1]Laboratory of Pharmacology, Faculty of Pharmaceutical Sciences, University of São Paulo, 14049-903 Ribeirão Preto, S.P. Brazil; and [2]University of Manchester, Rheumatic Diseases Centre, Hope Hospital, Salford, M6 8HD, UK

[CA] Corresponding Author

**The aim of this study was to investigate the pyrogenic activity of factor(s) released by rat peritoneal macrophages following a brief stimulation with LPS. The effect of this factor on the number of circulating leukocytes and serum Fe, Cu and Zn levels, was also evaluated. The possibility that the content of interleukin (IL)-1β, IL-6 and tumour necrosis factor (TNF) in the supernatant could explain the observations was investigated. Supernatant produced over a period of 1 h by peritoneal macrophages, following a 30 min incubation with LPS at 37°C, was ultrafiltered through a 10 000 MW cut-off Amicon membrane, sterilized, and concentrated 2.5, 5, 10 and 20 times. The intravenous (i.v.) injection of this supernatant induced a concentration-dependent fever in rats with a maximal response at 2 h. The pyrogenic activity was produced by macrophages elicited with thioglycollate and by resident cells. The supernatants also induced neutrophilia and reduction in Fe and Zn 6 h after the injection. Absence of activity in boiled supernatants, or supernatants from macrophages incubated at 4°C with LPS, indicates that LPS was not responsible for the activity. *In vitro* treatment with indomethacin (Indo), dexamethasone (Dex), or cycloheximide (Chx) did not modify the release of pyrogenic activity into the supernatant or its effects on the reduction in serum metal levels. Although Chx abolished the production of mediator(s) inducing neutrophilia, and Dex reduced the induction of IL-1β, TNF and IL-6, injection of the highest concentration of these cytokines detected in the supernatants did not induce fever. *In vivo* treatment with Dex, but not Indo, abolished the fever induced by the supernatant. These results suggest that macrophages contain pre-formed pyrogenic mediator(s), not related to IL-1β, IL-6 or TNF, that acts indirectly and independently of prostaglandin. It also seems likely that the pyrogenic activity is related to the factor responsible for the reduction of serum Fe and Zn levels, but not the neutrophilia.**

**Key words:** Lipopolysaccharide, Macrophages, Pyrogenic factor

### Introduction

Exogenous pyrogens such as bacterial endotoxin (lipopolysaccharide, LPS) initiate a complex host response which includes fever, neutrophilia and changes in circulating glucocorticoid, glycoproteins and serum metal levels, collectively called the acute-phase reaction (APR). It is thought that LPS-induced APR is caused by the release of cytokines into circulation by several cell types including macrophages, endothelial cells and fibroblasts. Many of these cytokines, including interleukin-1 (IL-1), interleukin-6 (IL-6), tumour necrosis factor (TNF), interleukin-8 (IL-8) and macrophage inflammatory protein-1 (MIP-1), generally known as endogenous pyrogens (EP) can induce fever when injected into experimental animals and humans (see Reference 1 for a review). Although all endogenous pyrogens can induce fever the real role of each in the febrile response is not clearly established.[1]

It has been suggested that IL-1 (largely IL-1β) is a primary mediator of fever since peripheral or central injection of this cytokine induces fever in different species.[2–4] In addition, antiserum to IL-1β prevents the increase in body temperature and plasma IL-6 activity induced by intraperitoneal (i.p.) injections of LPS in rats.[5] However, there is little data demonstrating an elevation of IL-1β in the plasma of febrile animals and no increase in this cytokine was detected in the cerebrospinal fluid of cats[6] or in the hypothalamus of rats[7] after peripheral injection of LPS. An increased synthesis of IL-1β in the

© 1994 Rapid Communications of Oxford Ltd hypothalamus of rats was observed by using doses of endotoxin that in terms of fever induction are supra-maximal.[8]

Recent studies in guinea-pigs[9] and rats[7] have shown an increase in intrahypothalamic concentrations of IL-6- and TNF-like activity during LPS-induced fever. In spite of a strong correlation between plasma IL-6 activity and increase in the body temperature in guinea-pigs, there was no correlation between fever and plasma TNF, or between fever and IL-6 or TNF in the hypothalamic perfusate. In rats, the hypothalamic infusion of IL-6, but not TNF, at a rate that would mimic the amount of cytokine activity found in the anterior hypothalamus during LPS-induced fever, was effective in inducing an increase in body temperature.

Data from many studies suggest that the first cytokine to increase in plasma after LPS injection in different animal species is TNF[9–11] but its role in fever is still unresolved. Intravenous infusions of TNF in humans, or bolus injection of this cytokine in rats and other species, result in fever.[4,10,12–14] Furthermore, pre-treatment of rabbits with antiserum to TNF inhibits LPS-induced fever.[15] However, recent studies attribute to TNF the role of an endogenous cryogen, rather than an endogenous pyrogen, since antiserum to TNF has been shown to increase LPS-induced fever in rats.[16–19]

Interleukin-8 is a cytokine released by a wide variety of cell types upon exposure to inflammatory stimuli or endogenous mediators such as IL-1 and TNF[20–22] and has been shown to induce fever after central administration in rats. Unlike the response to other cytokines, the febrile response induced by IL-8 is not dependent on prostaglandin synthesis.[23,24]

Macrophages represent one of the main sources of known cytokines[25] and have also been shown to release a chemotactic factor that specifically induces neutrophil migration.[26] This chemotactic substance, macrophage-released neutrophil chemotactic factor (MNCF), is released by rat peritoneal macrophages following a brief *in vitro* stimulation with heterologous serum, LPS, TNFα or IL-1β.[27–30] This chemotactic factor is distinguishable from other known chemotactic agents/or cytokines[31] and acts directly.[30]

Based on the ability of macrophages to release a neutrophil chemotactic substance different from the known cytokines, and because of the uncertain role of known cytokines in the genesis of fever, the purpose of this study was to investigate whether this factor could induce fever and other APR components such as changes in circulating white blood cells and the concentration of serum metals. In addition, the concentration of IL-1β, TNF and IL-6 released by macrophages during a brief incubation with LPS was determined as well as the effect of *in vitro* and *in vivo* treatment with steroidal and non-steroidal anti-inflammatory drugs and protein synthesis blockers on the release and action of the factor(s).

### Material and Methods

*Animals*: Male Wistar rats weighing 180–200 g were housed at 24 ± 1°C with 12 h light/dark cycle, with lights on at 0600, and given water and food *ad libitum*.

*Preparation of the supernatant from LPS-stimulated macrophage monolayers*: The supernatant was prepared using a process similar to that described previously by Cunha and Ferreira.[29] Briefly, 4 days before the harvest of peritoneal macrophages, rats received an intraperitoneal injection of 10 ml of 3% thioglycollate. To evaluate possible effects of thioglycollate, an equal number of macrophages from non-injected animals were used in some experiments. Peritoneal cells were harvested, using 10 ml of RPMI 1640 medium (pH 7.4) containing 5 U/ml heparin, and incubated in culture dishes for 1 h at 37°C. To discard the non-adherent cells, the monolayers were washed three times with phosphate buffered saline (PBS). Adherent cells in two or three plates of each treatment were scraped using a rubber policeman, resuspended in media, diluted in Turk's stain, and counted in a Neubauer chamber. The differential cell count was performed as described previously by Souza and Ferreira[27] and cellular viability was tested using 1% eosin Y.[32] The monolayers consisted of 95% macrophages, resulting in a total of $0.6–1.0 \times 10^7$ viable cells/plate. The cells were incubated with LPS (10 μg of lipopolysaccharide *E. coli* 0111:B4/ml of RPMI) for 30 min at 37°C. The monolayers were then washed three times with PBS and incubated with 5 ml of medium without LPS, for 1 h at 37°C. To evaluate the effect of the incubation temperature on the release of substances, some experiments were conducted at 4°C after adherence of the cells. The supernatant was ultrafiltered with an Amicon YM 10 membrane, the retained portion was resuspended in the saline for intravenous injections or lyophilized and stored at −20°C for cytokine assays. To discount the possibility of contamination with LPS, supernatant (10 times concentrated) and LPS were boiled for 30 min. The supernatants were sterilized with a 0.22 μm (Millipore Corporation, Bedford) membrane prior to intravenous (i.v.) injections.

*Treatment of macrophage monolayers with dexamethasone, cycloheximide and indomethacin*: Macrophage monolayers were incubated with 3 or 9 μg/ml dexamethasone (Dex), 3 and 9 μg/ml cycloheximide (Chx) or 0.5 and 2 μg/ml indomethacin (Indo) for 60, 30 or 15 min respectively, before LPS addition. During and after LPS

stimulation, the monolayers were incubated with RPMI medium containing the same concentration of the drugs. Control supernatants had the drugs added at the end of the incubation period. The preparation of the supernatant for injection was carried out as described above.

*Temperature measurements*: Rectal temperature was measured by inserting a thermistor probe (Y.S.I. nr. 402) 3 cm into the rectum. The animals were picked up gently and held manually during the temperature measurements. This procedure was performed at least twice on the day before the experiment to minimize stress-induced temperature changes secondary to handling. On the day of the experiment, the basal temperature of each animal was determined four times, at 30 min intervals, before any injection. The experiments were carried out between 0800 and 1700 h at 28 ± 1°C, which is considered the thermoneutral zone for rats.[33]

*Leukocyte counts and serum metal assays:* Two, 6 and 24 h after the i.v. injection of pyrogenic stimuli, animals were anaesthetized with pentobarbitone sodium (40 mg/kg, i.p.) and blood samples were taken from the abdominal aorta for haematological determination. Total and differential cell counts were performed and the results are reported as the number of neutrophils per ml of blood.[27] Before the serum metal assays, serum was prepared by adding the same volume of trichloroacetic acid 20% and heating at 90°C for 15 min. The mixture was centrifuged at 1000 $g$ by 15 min and the Fe, Zn and Cu levels were measured in the supernatant using atomic absorption spectrophotometry.

*Experimental protocols:* The rectal temperature of the rats was measured at 30 min intervals for 2 h before, and up to 6 h after i.v. injections of pyrogens. LPS (0.5 μg/kg), murine recombinant IL-1β (2.5 μg/kg) or supernatants were administered intravenously in the penial venous sinus in a volume of 0.2 ml. The effects of steroidal and non-steroidal anti-inflammatory drugs on temperature responses to i.v. injections of the supernatant, LPS and IL-1β were also investigated. Indo (2 mg/kg, i.p.) and Dex (0.5 mg/kg s.c.) were given 30 min and 1 h, respectively, before the i.v. injection of supernatants. Control animals were treated with the appropriate vehicle only. The pyrogenic stimuli were injected at 1100 h.

*Cytokine assays*: Cytokine bioactivities were measured in supernatants from Dex-treated macrophages and control supernatants. For these studies, the supernatant was lyophilized and resuspended in culture medium. IL-1 was assayed as described previously, using the D10(N4)M (D10) cell line.[34] Activity was determined by comparison to the National Institute of Biological Standards and Controls (NIBSC) interim standard for IL-1β (lot. 86.552, 1 U ≃ 10 pg).

IL-6 was assayed using the B9 hybridoma[35,36] as described previously by Holt *et al.*[37] For the assay, B9 cells were used 3–4 days after the last subculture. Bioactivity was determined by comparison to a recombinant IL-6 preparation provided by Dr L. A. Aarden.[38]

TNF was assayed using L929 fibroblasts, using a protocol adapted from Matthews and Neale.[39] The cells were maintained in DMEM supplemented with 5% FCS and 2 mM glutamine. Cells isolated with 0.25% trypsin, were seeded into microplates wells at $1 \times 10^4$ cells/wells, and cultured overnight with 200 pg/ml actinomicin D for 1 h before addition of samples or controls. The TNF concentration was determined by comparison to the NIBSC standard for TNFα (lot. 88/532). Cellular activity in all assays was determined by monitoring metabolism of 3-(4,5-dimethylthiazol-2-yl)-2,5-diphenyltetrazolium bromide to its formazan product.[40]

*Materials:* Lipopolysaccharide from *E. coli* (0111:B4), cycloheximide, glutamine, trypsin and MTT were purchased from Sigma Chemicals and Co., USA; thioglycollate from Difco Laboratories, USA; RPMI from Gibco Laboratories, USA; heparin from Roche, Brazil; yellow eosin from Merck; dexamethasone (Decadronal) from Prodome, Brazil; and DMEM from Northumbria Biologicals. Indomethacin was a gift from Merck, Sharp & Dohme, Brazil. Recombinant murine cytokines used to induce fever were IL-1β (lot. No. BN091), IL-6 (lot. No. BE1N3.19) and TNFα (lot. No. CS184), all from R & D Systems, Inc., Minneapolis.

*Statistical analysis*: Febrile responses were assessed by measuring the increases in rectal temperature and are expressed as the difference between rectal temperature before and at specified times after injection of the pyrogenic stimuli. All data are expressed as the mean ± S.E.M. and were analysed using one-way analysis of variance, followed by Tukey's test, and a level of significance at $p < 0.05$.

### Results

Intravenous injection of supernatant from thioglycollate-elicited macrophages stimulated *in vitro* with LPS induced a concentration dependent fever as illustrated in Fig. 1A. The i.v. injection of 2.5-fold concentrated supernatant did not change the rectal temperature of the animals, while 5-, 10- and 20-fold concentrated supernatant induced a considerable febrile response which peaked at 2 h. Both 10- and 20-fold concentrated supernatant, induce a maximal response, although the response to the higher concentration declined more rapidly. The 20-fold concentrated supernatant was used in the remaining experiments. Induction of the macrophages

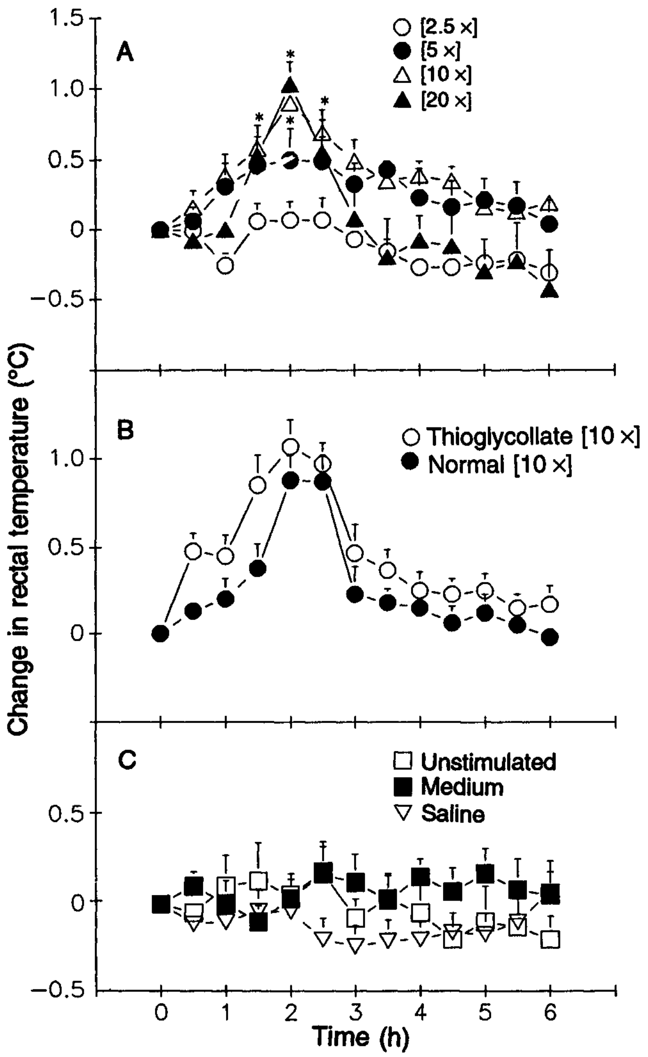

FIG. 1. Panel A: concentration dependent febrile response induced by supernatants from LPS-stimulated macrophages harvested from thioglycollate-elicited cavities. Panel B: febrile response induced by supernatant from thioglycollate or resident macrophages stimulated *in vitro* wit LPS. In both panel A and B macrophages were incubated *in vitro* with LPS for 30 min and supernatants were injected i.v. in concentrations indicated in the Figure. Panel C: control groups received supernatant from unstimulated macrophages, culture medium or saline. Each curve shows means ± S.E.M. of rectal temperature changes. Basal rectal temperatures (°C) were: Panel A: (O), 37.6 ± 0.10, *n* = 7; (●), 37.4 ± 0.18, *n* = 7; (△), 37.3 ± 0.10, *n* = 8; (▲), 37.8 ± 0.17, *n* = 4. Panel B: (O), 37.3 ± 0.06, *n* = 8; (●), 37.4 ± 0.07, *n* = 6. Panel C: (□), 37.3 ± 0.11, *n* = 7; (■), 37.5 ± 0.17, *n* = 4; (▽), 37.5 ± 0.11, *n* = 9. *Values significantly different from saline, $p < 0.05$.

with thioglycollate was not essential to the release of pyrogenic factor(s) since supernatant from LPS-stimulated resident macrophages induced fever with similar time-course and magnitude (Fig. 1B) when compared to the response induced by supernatant from LPS-stimulated macrophages previously elicited by thioglycollate. Injection of the medium, supernatant from unstimulated macrophages or saline, did not change the body temperature of the rats (Fig. 1C). LPS contamination was not responsible for the supernatant-induced fever since supernatant from LPS-stimulated macrophages cultured at 4°C did not induce fever. Furthermore, as shown in Table 1, no change in body temperature was observed after i.v. injection of supernatant boiled for 30 min. *In vitro* treatment of macrophages with 3 or 9 μg/ml Dex, 3 or 9 μg/ml Chx or 0.5 or 2.0 μg/ml Indo did not modify the pyrogenic activity of the supernatant (Fig. 2).

Supernatant from LPS-stimulated macrophages, harvested from thyoglycollate-elicited peritoneal cavities, induced a significant neutrophilia and reduction of the serum Fe level 2 and 6 h after i.v. injection, while reduction of serum Zn levels was evident only at 6 h. On the other hand, LPS caused neutrophilia 2 and 6 h after i.v. injection, while reduction of Fe and Zn levels was observed only at 6 h. In both cases the observed neutrophilia and reduction of serum metal levels were more marked at 6 h (Table 1). Neither the supernatant nor LPS induced any change of the serum Cu level. Boiling diminished the neutrophilia, abolished the reduction of the Zn level, but did not modify the reduction of the Fe level induced by the supernatant (Table 1).

**Table 1.** Changes in body temperature, circulating neutrophils, serum Fe and Zn levels induced by saline, LPS, non-boiled and boiled supernatants from LPS-stimulated macrophages in rats

| Pyrogenic stimulus | Time (h) | Temperature change (°C) | Neutrophils (cells/ml.10$^{-6}$) | Fe (μg/100ml) | Zn (μg/100ml) |
|---|---|---|---|---|---|
| Saline | 2 | 0.02 ± 0.10 | 1.68 ± 0.18 | 240.0 ± 23.7 | 348.4 ± 20.2 |
| (1 ml/kg) | 6 | 0.00 ± 0.10 | 1.17 ± 0.14 | 133.3 ± 17.5 | 267.3 ± 11.5 |
| LPS | 2 | 1.14 ± 0.15* | 5.75 ± 0.37* | 223.8 ± 14.7 | 329.5 ± 11.9 |
| (0.5 μg/kg) | 6 | 0.13 ± 0.21 | 6.84 ± 0.77* | 19.4 ± 10.4* | 147.0 ± 23.1* |
| Non-boiled | 2 | 1.02 ± 0.06* | 3.13 ± 0.39* | 106.5 ± 7.8* | 311.7 ± 10.3 |
| supernatant (10 x) | 6 | −0.08 ± 0.05 | 9.10 ± 1.18* | 78.9 ± 14.1* | 202.1 ± 14.1* |
| Boiled | 2 | −0.10 ± 0.06** | ND | ND | ND |
| supernatant (10 x) | 6 | −0.43 ± 0.07 | 3.51 ± 0.44** | 16.7 ± 3.6** | 267.0 ± 16.0** |

Macrophages from thioglycollate-elicited peritoneal cavities were stimulated *in vitro* with LPS for 30 min. The supernatants were ultrafiltered with an Amicon YM-10 membrane and resuspended in saline. Non-boiled and boiled (30 min boiling period) supernatant was sterilized with a 0.22 μm membrane and injected i.v. in a volume of 0.2 ml/rat. Values represent the mean ± S.E.M. of each parameter observed in 6–13 animals. ND = Not done; *significantly different from saline; **significantly different from non-boiled supernatant, $p < 0.05$.

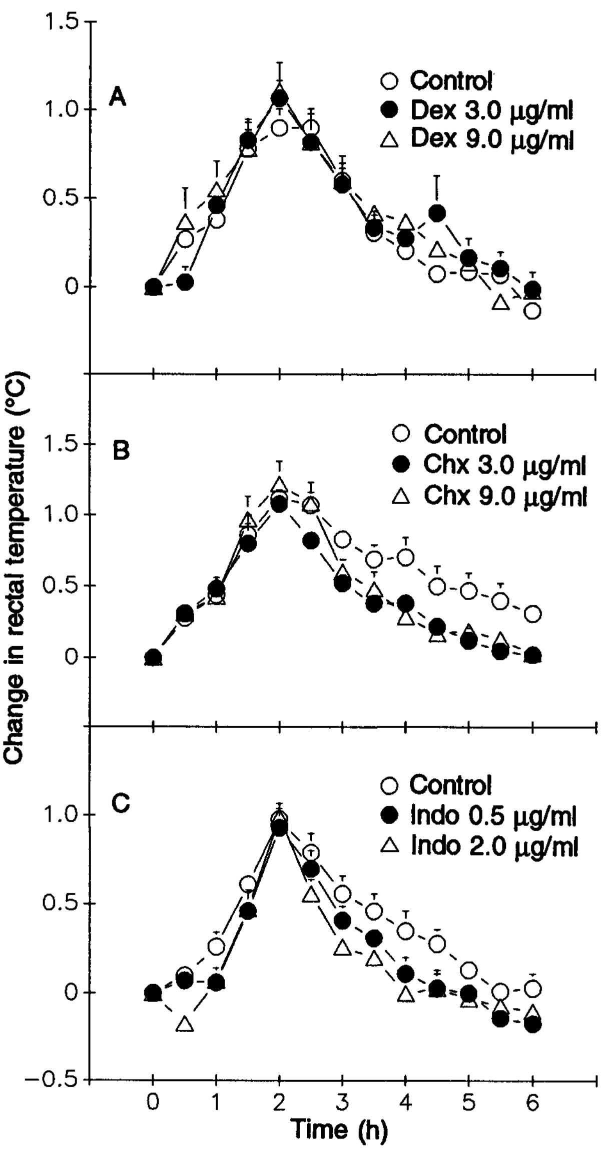

FIG. 2. Effect of Dex (Panel A), Chx (Panel B) and Indo (Panel C) upon release of pyrogenic factor(s) from LPS-stimulated macrophages. Macrophages were incubated with drugs before, during and after LPS stimulation. Control supernatant had the highest concentration of the drug added after the last incubation. The supernatants were injected as described in the legend to Fig. 1. Each curve shows mean ± S.E.M. of rectal temperature changes observed in 7–11 rats. Basal rectal temperatures (°C) were: Panel A: (○), 37.2 ± 0.08; (●), 37.3 ± 0.06; (△), 37.2 ± 0.05. Panel B: (○), 37.3 ± 0.05; (●), 37.3 ± 0.04; (△), 37.3 ± 0.03. Panel C: (○), 37.3 ± 0.06; (●), 37.2 ± 0.04; (△), 37.3 ± 0.05.

The neutrophilia, but not the reduction of serum metal levels, induced by the supernatant was significantly reduced when the macrophages were incubated in the presence of Chx (Fig. 3). The treatment of the macrophages with Dex or Indo did not significantly affect the neutrophilia induced by the supernatant 6 h after the injection (control supernatant, 6.47 ± 1.05; Dex, 9 μg/ml, 4.05 ± 0.72; Indo, 2 μg/ml, 8.45 ± 1.17 neutrophils × $10^6$/ml). When rats were pretreated with Dex (0.5 mg/kg, s.c.) 1 h before the harvesting of the peritoneal macrophages and all further steps of the supernatant preparation were conducted in presence of 9 μg/ml Dex, the magnitude of fever induced by the resulting supernatant was similar to that induced by supernatant from macrophages obtained from untreated animals (Fig. 4). Treatment of rats with Dex (0.5 μg/kg, s.c.) inhibited febrile responses to i.v. injection of LPS, IL-1β and supernatant (Fig. 5). In contrast, Indo (2 mg/kg, i.p.) significantly reduced the febrile response to i.v. injections of LPS and IL-1β, but did not modify the response induced by the supernatant (Fig. 6). The treatment of animals with Dex and Indo did not affect the neutrophilia or the reduction of serum Fe and Zn levels induced by LPS or supernatant.

The IL-1β, IL-6 and TNF bioactivities were measured in supernatants. Values varied quite widely

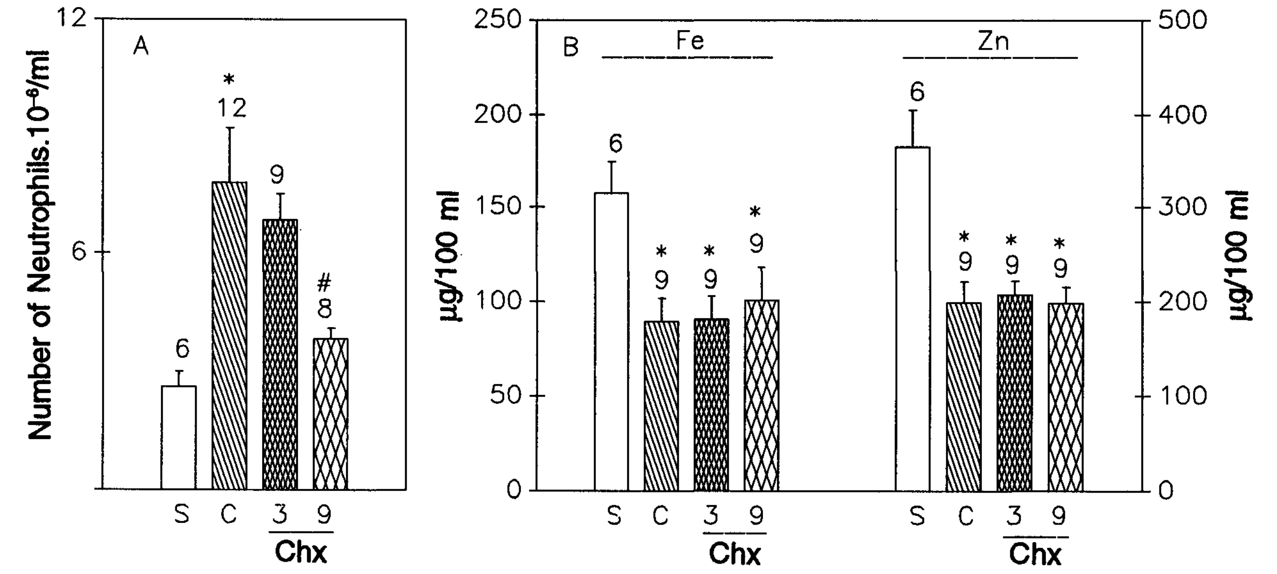

FIG. 3. Effect of *in vitro* treatment with Chx on the release of factor(s) from LPS-stimulated macrophages, that induce neutrophilia (Panel A) and changes in metal levels (Panel B). Blood samples were collected 6 h after the injections of supernatants from untreated (C) and treated with Chx (3 and 9 μg/ml) macrophages (Chx). Control groups received saline (S). The bars represent the mean ± S.E.M. of the neutrophil number or metal levels. The number of animals per group is indicated above each bar. *Values different from saline; #Values different from control supernatant, $p < 0.05$.

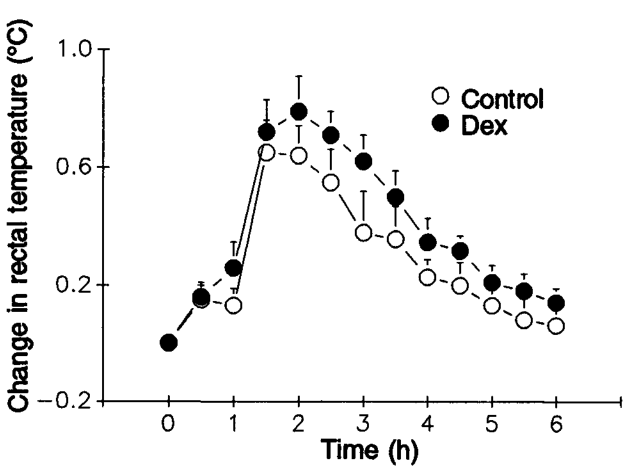

FIG. 4. Effect of *in vivo* and *in vitro* Dex treatment on release of pyrogenic factor by LPS-stimulated macrophages. Dex at 0.5 mg/kg was injected 1 h before harvesting of peritoneal macrophages. In all further steps of supernatant preparation, macrophages were treated with 9 μg/ml Dex. Control supernatant received Dex after the last incubation. Each curve shows mean ± S.E.M. of rectal temperature changes observed in 8–10 rats. Basal rectal temperatures (°C) were: (○), 37.15 ± 0.08; (●), 37.14 ± 0.04.

between different supernatants and the highest values are shown in Table 2. Unlike the pyrogenic activity Dex inhibited the release of these cytokines. The injection of the mixture of murine recombinant IL-1β, TNFα and IL-6 at the highest concentrations found in the supernatants induced a maximal increase in body temperature of 0.12 ± 0.04°C, 30 min after injection, which was not significantly different to saline treated controls (0.13 ± 0.03°C).

### Discussion

This study shows that macrophages briefly stimulated with LPS release a factor that induces an increase in rectal temperature, neutrophilia and reduction in serum Fe and Zn levels, mimicking important components of APR.

It is unlikely that these activities represent a non-specific response due to contamination of the supernatant with LPS since when the metabolic activity of the macrophages was reduced by cooling to 4°C, they did not release pyrogenic factor(s), and boiling the supernatant abolished the fever and Zn reduction and reduced the neutrophilia. The release of the pyrogenic factor(s) was not related to previous thioglycollate stimulation, since supernatant from LPS-stimulated resident macrophages also induced fever.

The treatment of the cells, or rats acting as the source of macrophages, with Dex did not modify the pyrogenic activity, although production of IL-1, IL-6 and TNF was inhibited. Since glucocorticoids suppress the cytokine or LPS-stimulated production of IL-1,[41] TNF,[42] IL-6, IL-8[43] and MNCF[29] these findings from *in vitro* Dex treatment distinguish the pyrogenic factor from other known pyrogenic or chemotactic

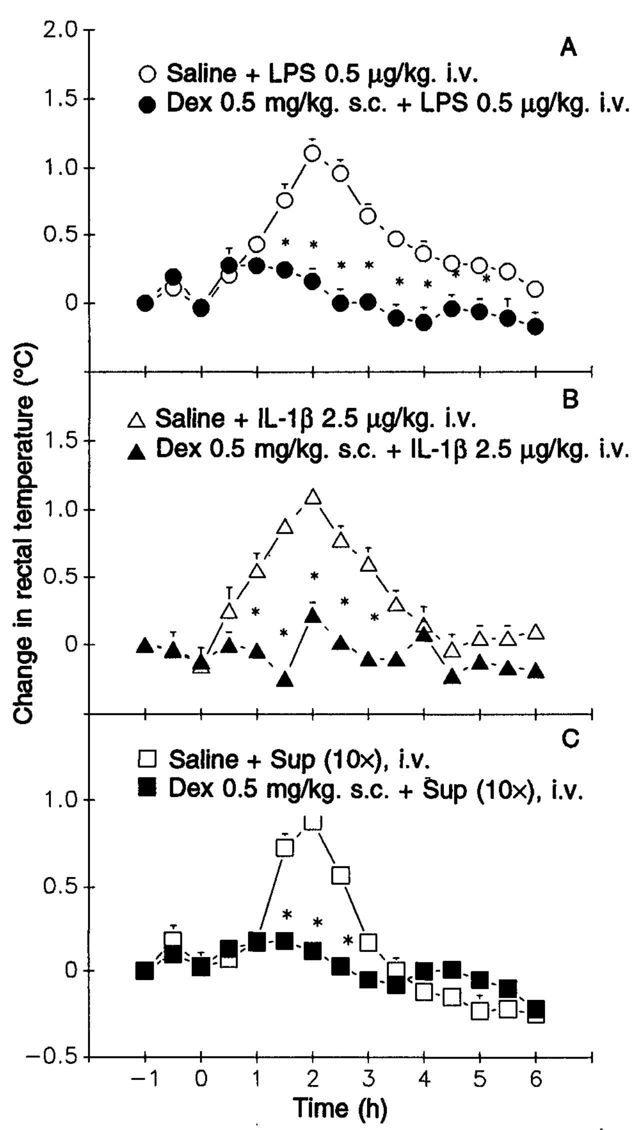

FIG. 5. Effect of treatment with Dex on the increases in rectal temperature induced by i.v. injection of LPS (Panel A), IL-1β (Panel B) or supernatant (Sup) from LPS-stimulated macrophages (Panel C). Animals were pretreated with 0.5 mg/kg s.c. Dex, 1 h before the injections of pyrogenic stimuli. Control groups were pretreated with saline. Each curve shows mean ± S.E.M. of rectal temperature changes observed in 5–8 rats. Basal rectal temperatures (°C) were: (○) 37.3 ± 0.06; (●), 37.2 ± 0.08; (△), 37.2 ± 0.06; (▲), 37.2 ± 0.05; (□), 37.0 ± 0.08; (■), 37.2 ± 0.09. *Significantly different from saline-treated animals, $p < 0.05$.

cytokines. In contrast, treatment of rats with Dex (0.5 mg/kg) inhibits fever induced by the supernatant or IL-1β.

The failure of Chx to inhibit the release of the factor that induces fever and reduction of the serum metal levels supports the hypothesis that this factor is not produced following synthesis of cytokines, though it did abolish the release of the factor that induced neutrophilia. Moore *et al.*[44] and Nordlund *et al.*[45] showed that Chx inhibits the production of EP by rabbit granulocytes or human monocytes stimulated with LPS or *Staphylococcus albus*. However, this inhibition was related to activity observed 12–16 h after stimulation. Therefore, it is plausible that macrophages release pre-formed factor. Similar findings have been recently obtained by Won and

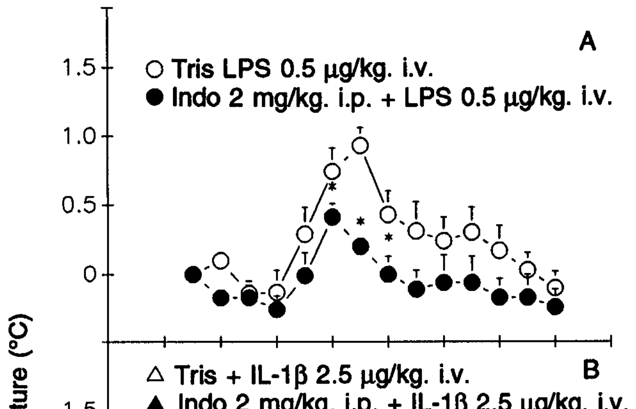

FIG. 6. Effect of treatment with Indo on the increase in rectal temperature induced by i.v. injection of LPS (Panel A), IL-1β (Panel B) or supernatant (Sup) from LPS-stimulated macrophages (Panel C). Animals were pretreated with 2 mg/kg, i.p., Indo 30 min before the injection of pyrogenic stimuli. Control groups received Tris buffer. Each curve shows mean ± S.E.M. of rectal temperature changes observed in 5–9 rats. Basal rectal temperatures (°C) were: (O), 37.4 ± 0.10; (●), 37.6 ± 0.13; (△), 37.1 ± 0.04; (▲), 37.2 ± 0.04; (□), 37.3 ± 0.04; (■), 37.3 ± 0.05. *Significantly different from Tris vehicle-treated animals, $p < 0.05$.

**Table 2.** Maximal concentration of IL-1β, IL-6 and TNFα in supernatants from LPS-stimulated macrophages treated or not with Dex.

| Treatment | IL-1β (ng/ml) | IL-6 (ng/ml) | TNFα (ng/ml) |
|---|---|---|---|
| None | 6.8 ± 0.45 | 10.17 ± 0.00 | 12.5 ± 0.23 |
| Dex | 2.3 ± 0.19* | 0.29 ± 0.2* | 2.3 ± 0.52* |

Macrophages were incubated with Dex (9 μg/ml) before, during and after LPS stimulation. The supernatants were ultrafiltered in YM-10 Amicon membranes, lyophilized and stored at –20°C until the day of the assay, when they were resuspended in culture medium to give a 10-fold concentrated solution. Values represent the mean ± S.E.M. (4–6 wells) cytokine concentration in the sample with the highest level of cytokine. *Values significantly different from untreated cells, $p < 0.001$.

Lin,[46] using human monocytes. In this study human monocytes, treated with Chx and stimulated with polyribo-inosinic : polyribocytidylic acid, released a pyrogenic factor into the supernatant. These results also show that different factors are responsible for the observed fever and neutrophilia.

It is unlikely that prostaglandins are responsible for the activities observed in the supernatant because the treatment with Indo did not modify its pyrogenicity or its ability to induce haematological changes (data not shown). The rapid inactivation after intravenous injections and the molecular weight (<10 kDa) of eicosanoids also make this unlikely. Although prostaglandins synthesized by monocytes/macrophages, inhibit the production of cytokines, including IL-1 and TNF,[47,48] Indo did not affect the pyrogenic activity of the supernatant.

Low concentrations of IL-1β, IL-6 and TNFα were found in the supernatant from LPS stimulated macrophages. Pretreatment of macrophages with Dex reduced these cytokine concentrations even more. However, the concentrations of cytokines necessary to induce fever of comparable magnitude to the supernatant are considerably higher.[49–51] A synergistic effect of these cytokines does not fully account for the observed febrile response, since the i.v. injection of a mixture of these cytokines, in the highest concentration found in the supernatants, did not induce any change in rectal temperature. The presence of cytokine precursors in the supernatant cannot be discounted, since precursors of IL-1α and β were found in supernatant from peritoneal macrophages after LPS stimulation.[52,53] On the other hand, the results obtained with Indo treatment suggest these precursors are not present because their active products induce a febrile response that is abolished by cyclooxygenase inhibitors.

Because our results point to some factor(s) different from the known pyrogenic cytokines we investigated the effect of anti-inflammatory drugs on the febrile response induced by the supernatant. Confirming previous results Dex inhibited the fever induced by LPS and IL-1β. Similarly, supernatant induced fever was inhibited by Dex, suggesting an indirect mechanism, e.g. the release of pyrogenic mediators. However, the treatment of animals with Indo did not modify the fever induced by the supernatant, while the febrile response induced by LPS and IL-1β was partially inhibited. Many studies have demonstrated that MIP-1 and IL-8 induce a prostaglandin-independent febrile response.[23,24,54] Thus, it is possible that these cytokines could be involved in supernatant induced fever.

Two factors suggest the supernatant activity is unlikely to be IL-8. Firstly, the relatively low concentration of other cytokines produced during 30 min of macrophage stimulation. Secondly, preliminary studies, using ultrafiltration in YM 30 membranes, have demonstrated that the MW of our pyrogenic factor is above 30 kDa. All the pyrogenic cytokines released by macrophages possess a molecular weight slower than 30 kDa.

Kluger[1] has stressed that although many cytokines induce fever when injected into experimental animals or humans, the precise contribution of any EP in naturally occurring fever has not yet been completely determined. This concept, together with evidence for a pre-formed pyrogenic factor described here, allow us to hypothesize that this factor may represent a trigger for fever induction by promoting the release of another pyrogenic mediator which acts by a prostaglandin independent mechanism. A possible candidate is CRF, explaining the effectiveness of glucocorticoids and the partial effectiveness of cyclooxgynase inhibitors in the febrile response induced in rats by IL-1β or LPS. These findings could explain the inability of antipyretic drugs to abrogate the fever response observed in a number of clinical situations. Furthermore, the present study emphasizes the importance of macrophages as alarm cells[55] because of their ability to react to foreign substances and release mediators responsible for the initial systemic and local events, such as fever, neutrophilia and cell migration.[30,56]

In conclusion, the results presented here allow us to suggest that macrophages contain pre-formed pyrogenic substance(s) which are not related to IL-1β, IL-6 or TNF, and act indirectly and independently of prostaglandin synthesis. It also seems likely that the pyrogenic activity is related to the factor responsible for the reduction of serum Fe and Zn levels, but not the neutrophilia.

### References

1. Kluger MJ. Fever: role of pyrogens and cryogens. *Physiol Rev* 1991; **71:** 93–127.
2. Long NC, Otterness I, Kunkel SL, Vander AJ, Kluger MJ. Roles of interleukin-1β and tumour necrosis factor in lipopolysaccharide fever in rats. *Am J Physiol* 1990; **259:** R724–R728.
3. Davidson J, Milton AS, Rotondo D. A study of the pyrogenic actions of interleukin-1α and interleukin-1β: interaction with a steroidal and non-steroidal anti-inflammatory agent. *Br J Pharmacol* 1990; **100:** 542–546.
4. Dinarello CA, Cannon JG, Mier JW, *et al.* Multiple biological activities of human recombinant IL-1. *J Clin Invest* 1986; **77:** 1734–1739.
5. LeMay LG, Otterness IG, Vander A, Kluger MJ. *In vivo* evidence that the rise in plasma IL-6 following injection of a fever-inducing dose of LPS is mediated by IL-1β. *Cytokine* 1990; *2:* 199–204.
6. Coceani F, Lees J, Dinarello CA. Occurrence of interleukin-1 in cerebrospinal fluid of the conscious cat. *Brain Res* 1988; **446:** 245–250.
7. Klir JL, Roth J, Szelenyi Z, McClellan JL, Kluger MJ. Role of hypothalamic interleukin-6 and tumor necrosis factor-α in LPS fever in rat. *Am J Physiol* 1993; **265:** R512–R517.
8. Hillhouse TW, Mosley K. Peripheral endotoxin induces hypothalamic immunoreactive interleukin-1β in the rat. *Br J Pharmacol* 1993; **109:** 289–290.
9. Roth J, Conn CA, Kluger MJ, Zeisberger E. Kinetics of systemic and intrahypothalamic IL-6 and tumor necrosis factor during endotoxin fever in guinea pigs. *Am J Physiol* 1993; **265:** R653–R658.
10. Michie HR, Spriggs DR, Kanogue KR, *et al.* Tumor necrosis factor and endotoxin induce similar metabolic responses in human beings. *Surgery St Louis* 1988; **104:** 280–286.
11. Remick DG, Strieten RM, Lynch III JP, Nguyen D, Eskandari M, Kunkel SL. *In vivo* dynamics of murine tumor necrosis factor-α expression. Kinetics of dexamethasone-induced suppression. *Lab Invest* 1989; **60:** 766–771.
12. Blatteis CM, Xim L, Quan N. Neuromodulation of fever: apparent involvement of opioids. *Brain Res Bull* 1991; **26:** 219–233.
13. Kettelhut IC, Goldberg AL. Tumour necrosis factor can induce fever in rats without activating protein breakdown in muscle or lipolysis in adipose tissue. *J Clin Invest* 1988; **81:** 1384–1389.
14. Rothwell NJ. Central effects of TNF-α on the thermogenesis and fever in the rat. *Biosci Rep* 1988; **8:** 345–352.
15. Nagai M, Saigusa T, Shimada Y, Inagana H, Oshima H, Iriki M. Antibody to tumor necrosis factor (TNF) reduces endotoxin fever. *Experientia Basel* 1988; **44:** 606–607.
16. Long NC, Kunkel SL, Vander AJ, Kluger MJ. Antiserum against TNF enhances LPS fever in the rat. *Am J Physiol* 1990; **258:** R332–R337.
17. Long NC, Vander AJ, Kunkel SL, Kluger MJ. Antiserum against tumor necrosis factor increases stress hyperthermia in rats. *Am J Physiol* 1990; **258:** R591–R595.
18. Mathinson JC, Wolfson E, Ulevitch RJ. Participation of tumor necrosis factor in the mediation of gram negative bacterial lipopolysaccharide-induced injury in rabbits. *J Clin Invest* 1988; **81:** 1925–1937.
19. Smith B, Kluger MJ. Normalization of body temperature and enhanced food intake in response to anti-tumor necrosis factor-α antibodies in cachectic tumor bearing rats. *Am J Physiol* 1993; **265:** R615–R619.
20. Baggiolini M, Walz A, Kunkel L. Neutrophil-activating peptide-1/interleukin 8, a novel cytokine that activates neutrophils. *J Clin Invest* 1989; **84:** 1045–1049.
21. DeForge LE, Kenney JS, Jones ML, Warren JS, Remick DG. Biphasic production of IL-8 in lipopolysaccharide (LPS)-stimulated human whole blood. *J Immunol* 1992; **148:** 2133–2141.
22. Van Zee KJ, DeForge LE, Fischer E, *et al.* Il-8 in septic shock endotoxemia, and after IL-1 administration. *J Immunol* 1991; **146:** 3478–3482.
23. Rothwell NJ, Hardwick AJ, Lindley I. Central actions of interleukin-8 in the rat are independent of prostaglandins. *Horm Metab Res* 1990; **22:** 595–596.
24. Zampronio AR, Souza GEP, Silva CAA, Cunha FQ, Ferreira SH. Interleukin-8 induces fever by a prostaglandin independent mechanism. *Am J Physiol* 1994; **266:** R1670–R1674.
25. Nathan CF. Secretory products of macrophages. *J Clin Invest* 1987; **79:** 319–326.
26. Cunha FQ, Souza GEP, Ferreira, SH. Macrophages stimulated with lipopolysaccharide release a selective neutrophil chemotactic factor: an *in vivo* demonstration. *Brazilian J Med Biol Res* 1986; **19:** 775–777.
27. Souza GEP, Ferreira SH. Blockade by antimacrophage serum of the migration of PMN neutrophils into the inflamed peritoneal cavity. *Agents and Actions* 1985; **17:** 97–103.
28. Souza GEP, Cunha FQ, Ferreira SH. Resident macrophages control initial neutrophil migration in the acute inflammatory response. In: Higgs GA, Williams TJ eds. *Inflammatory Mediators (Satellite symposia of the IUPHAR 9th International Congress of Pharmacology).* London: Macmillan Press, 1985; 149–156.
29. Cunha FQ, Ferreira SH. The release of a neutrophil chemotactic factor from peritoneal macrophages by endotoxin: inhibition by glucocorticoids. *Eur J Pharmacol* 1986; **129:** 65–76.
30. Faccioli LH, Souza GEP, Cunha FQ, Poole S, Ferreira SH. Recombinant interleukin-1 and tumor necrosis factor induce neutrophil migration *in vitro* by indirect mechanisms. *Agents and Actions* 1990; **30:** 344–349.
31. Dias-Baruffi M, Cunha FQ, Ferreira SH, Roque-Barreira MC. macrophage-released neutrophil chemotactic factor (MNCF) induces PMN-neutrophil migration through lectin-like activity. *Agents and Actions* 1993; **38:** Special Conference Issue.
32. Hanks JH, Wallace JH. Determination of cell viability. *Proc Soc Exp Biol Med* 1958; **98:** 188–192.
33. Gordon, CJ. Thermal biology of the laboratory rat. *Physiol & Behav* 1990; **47:** 963–991.
34. Hopkins SJ, Humphreys M. Simple, sensitive and specific bioassay of interleukin-1. *J Immunol Meth* 1989; **120:** 271–276.
35. Lansdorp PM, Aarden LA, Calafat J, Zeijlemaker WP. A growth-factor dependent B-cell hybridoma. In: Melchers F, Potter M, eds. *Current Topics in Microbiology and Immunology. Mechanisms in B-cell Neoplasia.* Berlin, Heidelberg: Springer-Verlag, 1986; 105–113.
36. Aarden LA, DeGroot ER, Schaap OL, Lansdorf PM. Production of hybridoma growth factor by human monocytes. *Eur J Immunol* 1987; **17:** 1411–1416.
37. Holt I, Cooper RG, Hopkins SJ. Relationships between local inflammation, interleukin-6 concentration and the acute phase protein response in arthritis patients. *Eur J Clin Invest* 1991; **21:** 479–484.
38. Brakenhoff JP, DeGroot ER, Evers RF, Pannenkock H, Aarden, LA. Molecular cloning and expression of hybridoma growth factor in *Escherichia coli. J Immunol* 1987; **139:** 4116–4121.
39. Mathews N, Neale ML. Cytotoxity assays for tumour necrosis factor and lymphotoxin. In: Clemens MJ, Morris AG, Gearing AJH, eds. *Lymphokines and Interferons: a practical approach.* Oxford, Washington DC: IRL Press, 1987; 221–225.
40. Tada H, Shimo O, Kuroshima K-I, Koyama M, Tsukamotto K. An improved colorimetric assay for interleukin-2. *J Immunol Meth* 1986; **93:** 157–165.
41. Staruch MJ, Wood DD. Reduction of serum interleukin-1-like activity after treatment with dexamethasone. *J Leukocyte Biol* 1985; **37:** 193–207.
42. Waage A, Bakke O. Glucocorticoids suppress the production of tumor necrosis factor by lipopolysaccharide-stimulated human monocytes. *Immunology* 1988; **63:** 299–302.
43. Tobler A, Meier R, Seitz M, Dewald B, Baggiolini M, Fey MF. Glucocorticoids down-regulate gene expression of GM-CSF, NAP-1/IL-8, and IL-6, but not of M-CSF in human fibroblasts. *Blood* 1992; **79:** 45–51.
44. Moore DM, Cheuk SF, Morton JD, Berlin RD, Wood Jr WB. Studies on the pathogenesis of fever. XVIII. Activation of leukocytes for pyrogen production. *J Exp Med* 1970; **131:** 179–188.
45. Nordlund JJ, Root RK, Wolff SM. Studies on the origin of human leukocytic pyrogen. *J Exp Med* 1970; **132:** 727–743.
46. Won SJ, Lin MT. Endogenous pyrogen formation by human blood monocytes stimulated by polyriboinosinic acid:polyribocytidylic acid. *Experientia* 1993; **49:** 157–159.
47. Knudsen PJ, Dinarello CA, Strom TB. Prostaglandins posttranscriptionally inhibit monocyte expression of interleukin-1 activity by intracellular cyclic adenosine monophosphate. *J Immunol* 1986; **137:** 3189–3194.
48. Hart PH, Whitty GA, Piccoli DS, Hamilton JA. Control by IFN-γ and $PGE_2$ of TNF-α and IL-1 production by human monocytes. *Immunol* 1989; **66:** 376–383.
49. Tocco-Bradley R, Moldawer LL, Jones CT, Gerson B, Blackburn GL, Bristian BR. The biological activity *in vivo* of recombinant murine interleukin-1 in the rat. *Proc Soc Exp Biol Med* 1986; **182:** 263–371.
50. Rothwell NJ. Mechanisms of the pyrogenic actions of cytokines. *Eur Cytokine Net* 1990; **1:** 211–213.
51. Bibby DC, Grimble RF. Temperature and metabolic change in rats after various doses of tumour necrosis factor α. *J Physiol* 1989; **410:** 367–380.
52. Suttles J, Giri JG, Mizel SB. IL-1 secretion by macrophages. Enhancement of IL-1 secretion and processing by calcium ionophores. *J Immunol* 1990; **144:** 175–182.
53. Beusher HU, Günter C, Röllinghoff M. IL-1β is secreted by activated murine macrophages as biologically inactive precursor. *J Immunol* 1990; **144:** 2179–2183.
54. Miñano FJ, Sancibrian M, Vizcaino M, *et al.* Macrophage inflammatory protein-1: unique action on hypothalamus to evoke fever. *Brain Res Bull* 1990; **24:** 849–852.
55. Ferreira SH. Are macrophages the body's alarm cells? *Agents and Actions* 1980; **10:** 229–230.
56. Souza GEP, Cunha FQ, Mello R, Ferreira SH. Neutrophil migration induced by inflammatory stimuli is reduced by macrophage depletion. *Agents and Actions* 1988; **24:** 377–380.

ACKNOWLEDGEMENTS. The authors gratefully acknowledge Márcio M. Coelho (from this department) for helpful discussions and review of the manuscript, and Saskia Brouwer for technical assistance. The Conselho Nacional de Desenvolvimento Científico e Tecnológico and Fundação de Amparo à Pesquisa do Estado de São Paulo is thanked for financial support (Proc. nr 500077/90 and 92/2012-8, respectively) and a fellowship to AR Zampronio and MCC Melo.

**Received 13 April 1994; accepted in revised form 8 June 1994**
